# Mechanistic domains in canine corneal ulcer progression: a conceptual framework for adjunctive management

**DOI:** 10.3389/fvets.2026.1825605

**Published:** 2026-06-19

**Authors:** Takuya Yogo

**Affiliations:** Department of Veterinary Surgery, Nippon Veterinary and Life Science University, Musashino, Japan

**Keywords:** anticollagenase therapy, canine corneal ulcer, keratomalacia, matrix metalloproteinase, regenerative ophthalmology, tear film instability

## Abstract

**Background:**

Canine corneal ulcers are commonly encountered in clinical practice and may progress to stromal melting and perforation if not appropriately managed. Adjunctive therapy has traditionally focused on collagenolysis inhibition and is often guided by morphological severity rather than dominant biological drivers. While anticollagenase strategies remain essential, ulcer progression involves interconnected processes, including inflammatory amplification, impaired epithelial repair, and ocular surface instability. This conceptual analysis therefore seeks to develop a mechanism-based conceptual framework that prioritizes these biological processes and guides adjunctive therapeutic decision-making.

**Methods:**

A targeted literature search was conducted using PubMed and Scopus for studies published between January 2000 and February 28, 2026. Relevant canine, animal model, and human studies were narratively synthesized to identify key pathophysiologic drivers and construct a mechanism-based hierarchical framework for adjunctive therapy. Interventions were categorized according to primary mechanistic targets and levels of supporting canine evidence.

**Results:**

The proposed framework delineates four therapeutic domains (Structural Preservation, Inflammatory Modulation, Regenerative Augmentation, and Functional Optimization) together with a meta-level component termed Precision Stratification. Structural Preservation, centered on protease inhibition, represents the highest biological priority in cases of active stromal melting. Inflammatory Modulation targets upstream amplification processes, whereas Regenerative Augmentation and Functional Optimization address epithelial repair and ocular surface stability, respectively. Precision Stratification is conceptualized as a meta-level decision-support component that aligns domain prioritization with dominant biological drivers. Evidence strength varies across domains; structural preservation strategies are supported by the broadest canine clinical evidence, whereas inflammatory modulation, regenerative, and precision-oriented strategies rely predominantly on translational or investigational data.

**Conclusion:**

The principal contribution of this work is the development of a mechanism-based hierarchical framework for adjunctive management of canine corneal ulcers. The framework preserves the central role of protease inhibition while situating it within a broader biologically structured model of corneal ulcer progression. Canine-specific validation of domain-targeted interventions and development of objective stratification tools represent important priorities for future investigation.

## Introduction

Canine corneal ulcers are among the most frequently encountered ophthalmic conditions in small animal practice and represent a common cause of ocular pain, vision impairment, and ophthalmic referral (1). Although many cases respond to conventional antimicrobial and supportive therapy, a subset progresses to stromal melting, descemetocele formation, or perforation, potentially resulting in permanent vision impairment (1). The biological processes underlying this progression are multifactorial and involve interactions among proteolytic enzymes, inflammatory mediators, epithelial repair mechanisms, and ocular surface stability (1, 2).

Adjunctive treatment strategies have traditionally focused on suppressing collagenolysis. Agents such as EDTA, autologous serum, and tetracyclines have contributed substantially to reducing stromal degradation and remain essential components of clinical management (1, 3). However, stromal dissolution represents only one aspect of a broader inflammatory and tissue-remodeling cascade. Cytokine signaling, neutrophil recruitment, oxidative stress, and impaired epithelial migration may also influence disease trajectory (4–6). Recognition of these interconnected pathways suggests that corneal ulcer progression cannot be explained solely by protease activity (4–6).

In clinical practice, therapeutic decisions are often guided by morphologic features such as ulcer depth, stromal involvement, and the presence of stromal melting. Although this approach is practical and widely accepted, these morphologic descriptors do not always reflect the dominant biological processes driving disease progression. Consequently, adjunctive therapies are often introduced empirically rather than selected according to the predominant pathophysiologic mechanism.

Recent developments in corneal immunobiology and regenerative research, including investigations into platelet-rich plasma and tear film biomarkers, have provided additional insight into mechanisms that may influence corneal healing dynamics (5, 7). Although canine-specific evidence remains heterogeneous and, in some areas, limited, many inflammatory and reparative pathways are conserved across mammalian species (5, 6). This biological continuity supports cautious translational synthesis while acknowledging existing evidence gaps.

The objective of this review is not only to summarize adjunctive therapies for canine corneal ulcers but also to develop a mechanism-based conceptual framework that prioritizes biological processes driving disease progression and healing.

**Figure 1:**
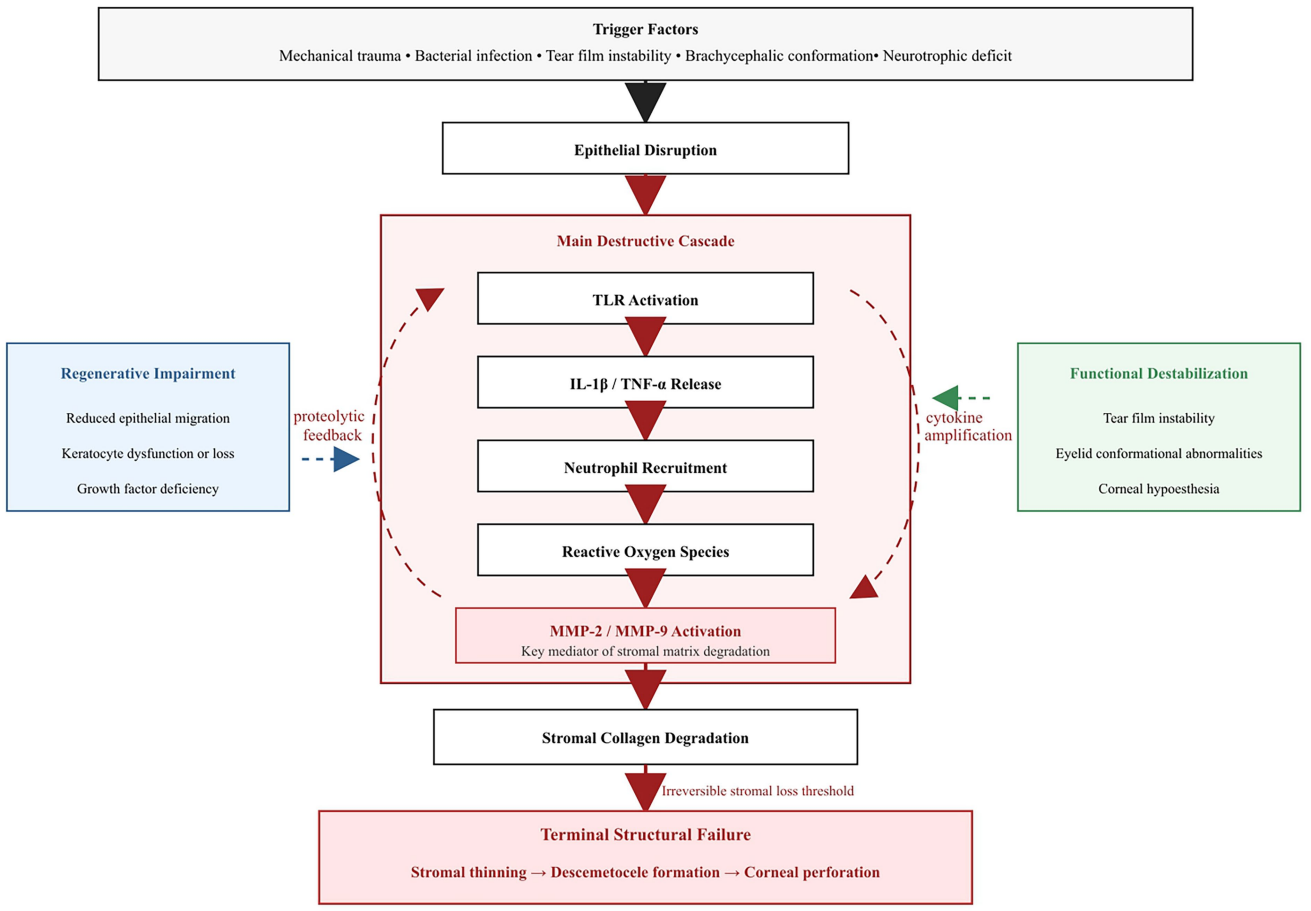
Mechanistic network underlying the progression of canine corneal ulcers. Schematic overview of the interacting biological processes involved in canine corneal ulcer progression. Etiologic triggers such as mechanical trauma and bacterial infection, together with predisposing factors including tear film instability, brachycephalic conformation, and neurotrophic deficits, can initiate epithelial disruption and activation of innate immune pathways. Subsequent cytokine signaling promotes neutrophil recruitment, reactive oxygen species production, and increased matrix metalloproteinase activity, leading to stromal collagen degradation. Concurrent regenerative impairment and ocular surface instability may delay epithelial repair. These interacting processes can ultimately result in stromal thinning, descemetocele formation, or corneal perforation.

This conceptual analysis proposes a mechanism-based hierarchical framework for the adjunctive management of canine corneal ulcers. By organizing therapeutic strategies into biologically defined domains according to biological urgency and the potential irreversibility of tissue loss, the framework aims to support structured clinical reasoning and identify areas requiring further investigation. These interacting biological processes underlying ulcer progression, which provide the biological rationale for the proposed framework, are summarized schematically in Figure 1.

### Literature identification strategy

A targeted literature search was conducted using PubMed and Scopus to identify studies relevant to the pathophysiology and adjunctive management of canine corneal ulcers.

Search terms included combinations of “canine corneal ulcer,” “keratomalacia,” “matrix metalloproteinase cornea dog,” “corneal inflammation cytokine dog,” “platelet-rich plasma eye dog,” “tear film instability dog,” “neurotrophic keratitis dog,” and related keywords. Reference lists of key articles were manually screened to identify additional relevant studies. Given the limited availability of canine-specific mechanistic studies, human and experimental animal literature were included when biologically translatable. Literature searches were conducted for studies published between January 2000 and February 28, 2026.

**Figure 2:**
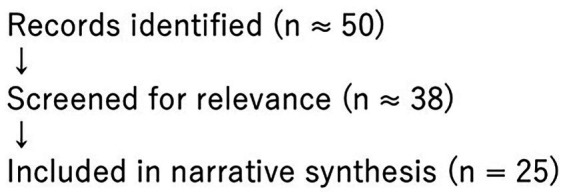
Simplified literature identification and selection process used for the targeted narrative synthesis. Approximately 50 potentially relevant studies were identified through database searching and manual screening of reference lists. After relevance-based screening focused on mechanistic and translational applicability to canine corneal ulcer pathophysiology and adjunctive management, 25 studies were included in the final narrative synthesis. Because this manuscript was developed as a targeted conceptual analysis informed by a structured narrative literature synthesis rather than a formal systematic review, approximate values are presented.

The targeted literature search identified approximately 50 potentially relevant articles, of which 25 studies were ultimately included in the narrative synthesis based on mechanistic relevance and translational applicability to canine corneal ulcer pathophysiology and adjunctive management. Studies lacking direct mechanistic relevance, canine translational applicability, or sufficient methodological detail were excluded. A simplified overview of the literature identification and selection process is provided in Figure 2. Literature screening and study selection were performed by a single reviewer (T. Y.).

### Inclusion philosophy

Studies were included if they (i) investigated molecular or cellular mechanisms relevant to stromal degradation, inflammation, or corneal healing; (ii) reported clinical outcomes of adjunctive therapies in dogs; and/or (iii) provided experimental evidence with translational relevance to canine corneal biology. Randomized controlled trials, case series, mechanistic studies, and experimental injury models were all considered. Conference abstracts lacking sufficient methodological detail and non–peer-reviewed sources were excluded.

### Narrative synthesis approach

Given the heterogeneity of study designs and the limited availability of randomized trials in veterinary ophthalmology, a formal meta-analysis was not feasible. Instead, a narrative synthesis approach was used to summarize mechanistic pathways contributing to ulcer progression, identify biologically distinct therapeutic targets, and organize adjunctive interventions according to shared pathophysiologic drivers. This approach prioritizes biological plausibility and mechanistic coherence over statistical aggregation.

### Framework construction process

Following literature synthesis, therapeutic strategies were organized into mechanistically defined domains based on the primary biological process targeted, the temporal urgency of the process in disease progression, and the degree of reversibility of tissue damage. Domains were ordered according to biological priority and time-criticality rather than therapeutic popularity or evidence volume. A concentric model was developed to visually represent the hierarchical structure of these intervention domains.

### Evidence level classification

To contextualize the strength of the supporting data, interventions were categorized as high, moderate, or low evidence. High evidence indicates multiple canine clinical studies with consistent findings. Moderate evidence indicates limited canine-specific studies supported by translational experimental or human data. Low evidence refers to interventions primarily extrapolated from non-canine data with limited direct validation. This classification is heuristic and is intended to provide transparency regarding translational confidence rather than to function as a formal evidence-based guideline (Table 1).

**Table 1:** Heuristic evidence classification used in the proposed mechanistic framework for adjunctive management of canine corneal ulcers.

Evidence level	Characteristics	Representative references
High	Multiple canine clinical studies with consistent findings	(1–3, 9, 12–14, 28, 29)
Moderate	Limited canine evidence supported by translational or experimental studies	(4–8, 10, 11)
Low	Primarily extrapolated from non-canine or emerging technologies	(15–18, 20, 25)

Evidence levels were assigned according to the relative amount of canine-specific clinical evidence, consistency of reported findings, and degree of reliance on translational or experimental data. This classification is intended to provide conceptual transparency within the narrative framework and does not represent a formal evidence-based grading system.

### Framework overview

Because the present framework focuses specifically on adjunctive interventions that modulate biological processes involved in ulcer progression, etiologic therapies such as antimicrobial treatment for infectious keratitis are not classified as independent domains within the model. Instead, these therapies are regarded as essential etiologic interventions that operate alongside the proposed adjunctive domains.

**Figure 3:**
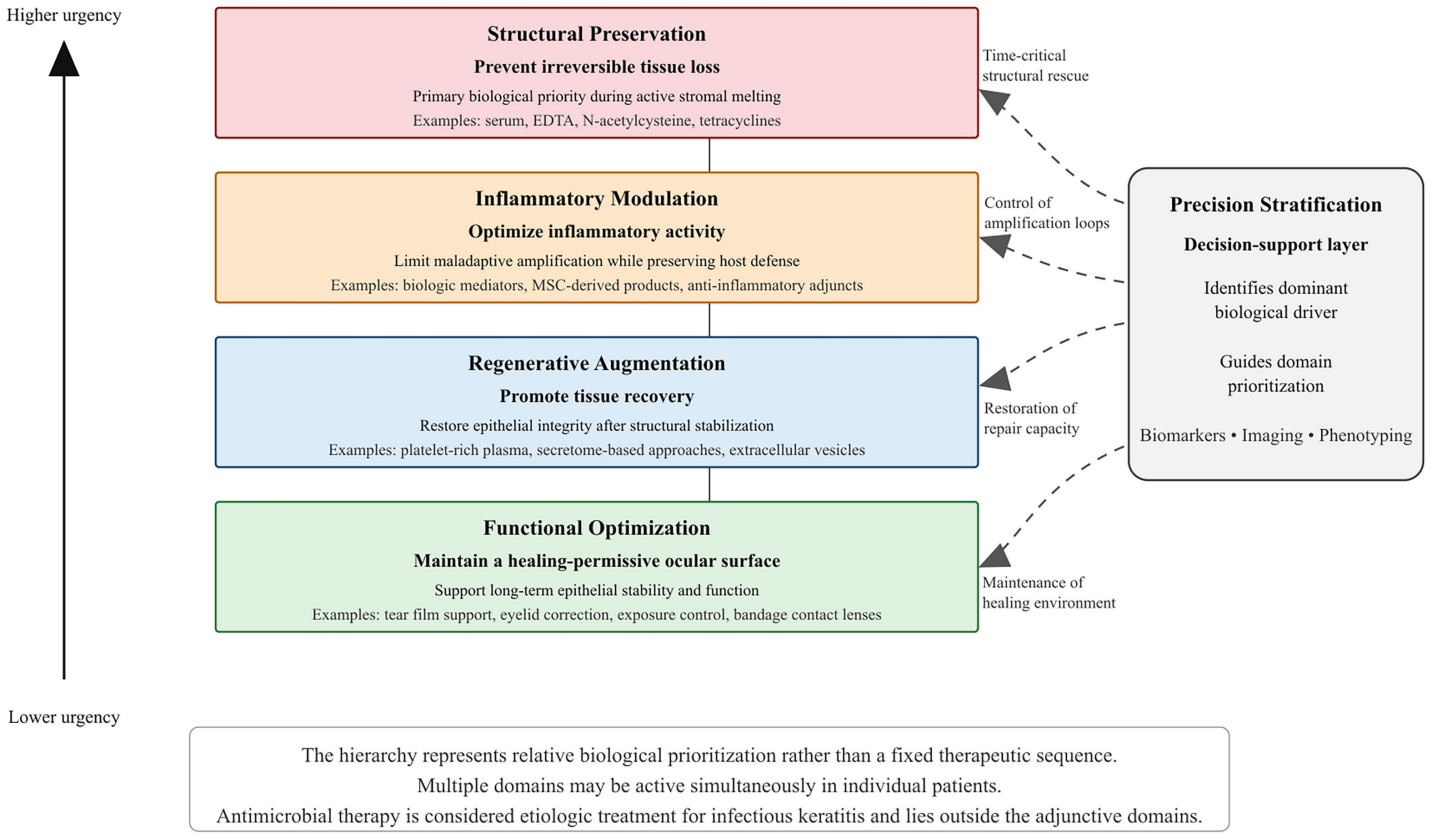
Conceptual framework for mechanism-based adjunctive management of canine corneal ulcers. The proposed framework organizes adjunctive interventions according to biological urgency, reversibility, and dominant pathophysiologic driver. Structural Preservation is prioritized as the highest-priority domain during active stromal melting because its primary objective is prevention of irreversible tissue loss. Inflammatory Modulation, Regenerative Augmentation, and Functional Optimization are distinguished by their primary conceptual roles: optimization of maladaptive inflammatory amplification, promotion of tissue recovery, and maintenance of a healing-permissive ocular surface, respectively. Precision Stratification is presented as a meta-level decision-support layer that may guide domain prioritization by identifying the dominant biological driver in an individual patient rather than a direct therapeutic intervention. The hierarchy is dynamic, and multiple domains may be relevant in the same patient. The hierarchy represents relative biological prioritization rather than a fixed therapeutic sequence. The outward progression from the central domain toward the peripheral domains reflects decreasing biological urgency and increasing emphasis on tissue restoration, ocular surface maintenance, and long-term functional outcomes rather than chronological disease progression.

Based on the synthesis of mechanistic and clinical literature, adjunctive management of canine corneal ulcers can be organized into biologically defined therapeutic domains that differ in urgency, reversibility, and pathophysiologic focus. Rather than representing independent or competing strategies, these domains describe interconnected levels of intervention that correspond to dominant biological drivers within the ulcerative process. The proposed biologically oriented therapeutic hierarchy, organized according to urgency, reversibility, and dominant pathophysiologic drivers, is illustrated schematically in Figure 3.

The proposed framework recognizes that corneal ulcer progression is governed by a dynamic network of interacting mechanisms, including proteolytic activity, inflammatory amplification, impaired regeneration, and ocular surface instability (1, 4–6). However, these processes do not contribute equally to disease progression. Certain events, particularly rapid stromal dissolution, are time-critical and may lead to irreversible structural loss if not promptly controlled (1). Other processes, such as delayed epithelial migration or tear film dysfunction, influence healing trajectory but typically operate over a longer timescale.

The innermost domain, Structural Preservation, focuses on the immediate control of stromal degradation. Its primary objective is to prevent progression to descemetocele formation or perforation by limiting excessive proteolysis. Stromal loss is largely irreversible; therefore, this domain represents the highest biological priority when active corneal melting is present.

Surrounding this core is the Inflammatory Modulation domain, which targets cytokine signaling, neutrophil recruitment, and related amplification pathways, including inflammasome signaling and neutrophil extracellular trap (NET) biology (4–8).

The Regenerative Augmentation domain focuses on the restoration of epithelial integrity and stromal architecture once acute structural collapse has been controlled. This domain includes biologically active blood products and emerging cell-free strategies (e.g., secretome−/exosome-based approaches) designed to provide regenerative and immunomodulatory cues (9–11).

The Functional Optimization domain addresses tear film instability, eyelid abnormalities, exposure-related factors, and neurotrophic deficits that predispose to delayed healing or recurrence (1, 2, 12–14).

Finally, Precision Stratification is conceptualized as a meta-level component of the framework that guides therapeutic selection across the other domains. Biomarker assessment, tear proteomic profiling, imaging-based phenotyping, and emerging machine-learning tools may assist in identifying dominant biological drivers in individual cases (15–18).

The proposed hierarchy does not represent a rigid therapeutic sequence or evidence-ranking system. Instead, it reflects relative biological priority based on the urgency, reversibility, and dominant pathophysiologic drivers contributing to corneal ulcer progression.

### Structural preservation domain

The Structural Preservation domain encompasses interventions aimed at preventing irreversible stromal loss by controlling excessive proteolysis. In cases of active stromal melting, this domain assumes the highest biological priority because stromal degradation can progress rapidly to descemetocele formation or perforation, and lost stromal tissue is largely irreversible.

Proteolytic activity in melting ulcers is mediated by host-derived matrix metalloproteinases, neutrophil elastase, and, in some cases, bacterial collagenases. Increased expression and activation of MMP-2 and -9 have been documented in the tear film of dogs with infectious keratitis, supporting the biological relevance of protease-driven stromal dissolution (1). These enzymes directly degrade stromal collagen fibrils and contribute to tissue softening and thinning (2, 3).

Anticollagenase therapies, including N-acetylcysteine, autologous serum, topical EDTA, and systemic or topical tetracyclines, therefore represent foundational interventions for melting ulcers (1, 3). N-acetylcysteine and autologous serum are widely used in clinical practice because of their accessibility and favorable safety profiles. EDTA reduces metalloproteinase activity through divalent cation chelation but may be associated with concentration-dependent epithelial toxicity. Clinical experience and published case series suggest that timely administration of these agents reduces the risk of progression to corneal perforation (1).

Within the proposed hierarchy, these therapies are necessary for structural stabilization but are not sufficient to address upstream inflammatory amplification or impaired epithelial repair. Their priority derives from the time-critical and irreversible nature of stromal loss rather than from exclusivity of mechanism. This positioning preserves their central clinical role while clarifying the need for complementary interventions from other domains to support preservation of corneal integrity and functional healing in complex cases.

### Inflammatory modulation domain

The Inflammatory Modulation domain addresses the amplification processes that sustain and intensify stromal degradation. Although proteolysis directly mediates collagen breakdown, it frequently occurs within a broader inflammatory cascade characterized by cytokine signaling, leukocyte recruitment, and oxidative stress (4–6). In this context, matrix degradation may represent both a primary destructive mechanism and a downstream consequence of inflammatory escalation.

Experimental and translational studies in non-canine models of corneal injury and microbial keratitis demonstrate that epithelial disruption exposes stromal tissue to damage- and pathogen-associated molecular patterns that activate innate immune pathways, including Toll-like receptor signaling (4–6). Subsequent production of interleukin-1β (IL-1β) and tumor necrosis factor-*α* promotes neutrophil recruitment and further upregulation of matrix metalloproteinases (4–6). Recent work has highlighted the role of inflammasome signaling, particularly NLRP3, as a regulatory hub linking pathogen recognition to IL-1β maturation and amplification of corneal inflammation (4–6). In parallel, NET formation has emerged as a double-edged component of antimicrobial defense that can contribute to tissue injury, angiogenic signaling, and sustained inflammatory activation (7, 8). Together, these processes form a self-reinforcing loop in which inflammation and proteolysis potentiate one another.

In canine corneal ulcers, detailed molecular characterization of upstream cytokine networks remains limited. However, the consistent presence of neutrophilic infiltration and elevated protease activity in melting lesions is compatible with inflammatory amplification mechanisms described in other species (2, 4–6). This cross-species consistency supports cautious translational inference while emphasizing the need for controlled canine-specific investigation.

Therapeutic strategies within this domain aim to attenuate maladaptive inflammatory escalation without compromising essential host defense. Experimental and translational studies have explored the immunomodulatory properties of mesenchymal stem cell (MSC)-derived products and related biologic mediators that influence cytokine production and leukocyte behavior (10, 11, 19). However, clinical data in dogs remain limited to exploratory reports and small case series, and standardized protocols have not been established (10, 11). Potential risks include variability in secretome composition, lack of standardized manufacturing protocols, safety considerations, and uncertainty regarding optimal timing and dosing strategies (10, 11).

Within the proposed framework, inflammatory modulation is prioritized when persistent stromal softening or marked inflammatory infiltration suggests ongoing escalation beyond isolated protease activity (4–8). It is therefore conceptualized as complementary to structural preservation strategies rather than as a replacement for protease inhibition. The objective is selective attenuation of amplification pathways that perpetuate tissue damage, rather than broad immunosuppression.

By distinguishing inflammatory escalation from direct matrix degradation, the framework clarifies why inflammatory modulation warrants consideration as a separate therapeutic domain. Inflammatory responses serve both protective and reparative functions; excessive amplification may accelerate stromal destruction, whereas inadequate inflammatory activity may impair pathogen control and tissue repair. Accordingly, the objective of this domain is not suppression of inflammation per se, but selective modulation of amplification pathways that support tissue preservation and subsequent healing while maintaining essential host defense.

### Regenerative augmentation domain

The Regenerative Augmentation domain addresses impaired epithelial repair and stromal remodeling that may persist after acute structural destabilization has been controlled. While inhibition of proteolysis is essential to prevent irreversible tissue loss, restoration of corneal integrity ultimately depends on effective re-epithelialization and coordinated stromal healing.

Corneal epithelial migration, adhesion, and proliferation are regulated by growth factors, extracellular matrix interactions, and keratocyte-mediated stromal signaling (20, 21). In chronic or indolent ulcers, epithelial healing may remain delayed despite stabilization of stromal degradation. In dogs, this delay may reflect insufficient trophic signaling and abnormalities of the epithelial basement membrane that impair durable epithelial adhesion (1, 10, 11, 22, 23). Persistent epithelial adhesion abnormalities and recurrent epithelial instability, as recognized in spontaneous chronic corneal epithelial defects (SCCEDs), may likewise interfere with stable re-epithelialization despite adequate biological support (22–24).

Conditions such as spontaneous chronic corneal epithelial defects illustrate how defective hemidesmosomal attachment and altered basement membrane architecture can prevent stable re-epithelialization despite ongoing epithelial migration (20, 21). Experimental injury models in multiple species demonstrate that biologically active mediators, including platelet-derived growth factors and secretome-associated cytokines, can accelerate epithelial closure and influence stromal remodeling dynamics (10, 11).

Emerging regenerative therapies may exert effects that extend beyond direct stimulation of epithelial repair. Experimental studies suggest that mesenchymal stem cell-derived products and extracellular vesicles can influence the local immune microenvironment through modulation of cytokine signaling, regulation of inflammatory cell recruitment, and promotion of pro-repair macrophage phenotypes, thereby linking regenerative and immunomodulatory processes during corneal healing (19, 20, 25).

Beyond epithelial closure, regenerative strategies may also influence stromal remodeling and transparency. In translational systems, MSC-derived products have been associated with modulation of myofibroblast differentiation and reduced corneal haze, although these effects remain insufficiently characterized in canine corneal disease (10, 11).

Within this framework, MSC-derived products are conceptualized primarily within the Regenerative Augmentation domain while acknowledging partial overlap with inflammatory modulation pathways.

In dogs, platelet-rich plasma has been investigated as a clinically applicable source of concentrated growth factors and bioactive mediators, with reported improvements in the healing of corneal ulcers (9). These blood-derived products represent a pragmatic regenerative strategy that is feasible in clinical practice. More recently, attention has shifted toward cell-free regenerative therapeutics, particularly MSC-derived secretomes and extracellular vesicles, which aim to retain immunomodulatory and trophic effects while improving standardization, storage, and safety profiles (10, 11). Although these approaches are biologically plausible and supported by an expanding translational literature, controlled comparative trials in canine corneal disease remain limited. Additional limitations include variability in preparation protocols and lack of standardization, cost considerations, and the absence of long-term controlled safety data in dogs.

Within the proposed framework, regenerative augmentation is prioritized once active stromal melting has been stabilized or when delayed epithelial healing becomes the dominant clinical feature. Its objective is not to replace structural preservation but to facilitate restoration of epithelial continuity and reduce the likelihood of chronicity or recurrence. By distinguishing ocular surface stability from proteolytic control and regenerative stimulation, the framework emphasizes that durable corneal healing requires not only arrest of tissue destruction and restoration of epithelial repair, but also maintenance of a microenvironment conducive to sustained healing. This domain is considered distinct because epithelial recovery depends not only on cellular repair mechanisms but also on tear film stability, sensory function, and ocular surface homeostasis. Even when tissue destruction is controlled and regenerative pathways are activated, persistent ocular surface dysfunction may compromise long-term healing and contribute to suboptimal functional outcomes (1, 2, 12–14). By distinguishing regenerative support from both protease inhibition and inflammatory modulation, this domain clarifies the role of bioactive adjuncts within a temporally structured treatment hierarchy. Regenerative interventions are therefore grouped separately because they primarily enhance tissue recovery rather than prevent tissue loss, although the strength of canine-specific evidence supporting these approaches remains moderate to low (9–11).

Although MSC-derived secretomes and extracellular vesicles are mechanistically attractive because they may promote epithelial repair, angiogenesis, extracellular matrix remodeling, and modulation of inflammatory signaling (19–21), their current translational status remains preliminary in veterinary ophthalmology. Available evidence is derived mainly from non-canine wound-healing models, human ocular surface studies, and early regenerative medicine literature rather than controlled canine corneal ulcer trials. Important practical barriers include variability in cell source, culture conditions, secretome or vesicle composition, delivery route, dosing schedule, storage conditions, production scalability, and cost. Therefore, these approaches should currently be regarded as investigational biologic adjuncts rather than established first-line therapies (10, 11, 19, 21).

Importantly, inflammatory modulation should not be viewed simply as the addition of another therapeutic modality but as a strategy directed toward the predominant biological process driving disease progression. In active melting ulcers, structural preservation remains the immediate priority because progressive collagen degradation threatens corneal integrity. However, when persistent inflammatory amplification appears to contribute substantially to ongoing tissue damage, modulation of inflammatory pathways may become an increasingly relevant adjunctive priority.

The proposed framework also encourages reassessment of the predominant pathological driver when healing fails to progress as expected. Rather than reflexively escalating anticollagenase therapy, clinicians should consider whether the principal biological bottleneck has shifted toward persistent inflammation, impaired epithelial repair, tear-film dysfunction, or mechanical instability. More broadly, the framework is intended to guide prioritization of biological processes rather than prescribe a fixed sequence of treatments, and the relative importance of individual domains may change throughout disease progression and healing.

### Functional optimization domain

The Functional Optimization domain addresses ocular surface stability as a determinant of epithelial integrity and sustained corneal healing. While this domain does not directly arrest acute stromal melting, it strongly influences healing trajectory, recurrence risk, and chronicity (1, 2, 12–14).

Ocular surface homeostasis depends on coordinated tear film production, lipid layer integrity, eyelid conformation, blink dynamics, and corneal sensory input. Disruption of any of these elements can impair epithelial adhesion, increase desiccation stress, and prolong ulcer duration despite adequate control of infection and proteolysis (1, 2).

Recent veterinary studies provide quantitative support for this domain. Measurements obtained using veterinary ocular surface analyzers have demonstrated that brachycephalic dogs exhibit significantly reduced tear film stability, reduced lipid layer thickness, and increased meibomian gland loss compared with non-brachycephalic breeds (12). These findings indicate that ocular surface instability may represent an intrinsic conformational predisposition rather than a mere secondary consequence of ulceration (12).

Similarly, comprehensive tear testing in brachycephalic dogs with and without keratoconjunctivitis sicca has indicated that reliance on Schirmer tear testing alone may underestimate functional instability, whereas combined qualitative and quantitative tear assessments better characterize surface dysfunction (13). These data reinforce the concept that tear film assessment should extend beyond aqueous deficiency when evaluating delayed epithelial healing.

Emerging evidence further suggests that neural regulation of tear production contributes to ocular surface resilience. Studies in dogs indicate that nasal sensory stimulation contributes to reflex lacrimation, and diminished reflex pathways in certain conformations may reduce adaptive tear responses (14). This highlights a neuro-functional component of ocular surface stability that may contribute to chronic epithelial vulnerability.

Clinically, correcting surface instability may include tear supplementation, management of meibomian gland dysfunction, surgical reduction of excessive palpebral fissure width, protection against exposure-related desiccation, and interventions aimed at supporting corneal sensory function (1, 12–14). Although randomized comparative trials remain limited, the convergence of quantitative ocular surface data and long-standing clinical observations supports functional optimization as a biologically distinct therapeutic domain.

Pharmacologic surface-stabilizing agents, including hyaluronic acid–based lubricants, mucin secretagogues such as diquafosol sodium, epithelial-protective agents such as rebamipide, and immunomodulatory tear-enhancing therapies such as topical cyclosporine, tacrolimus and pimecrolimus, may enhance tear film stability and epithelial resilience in predisposed dogs (26–30).

In immune-mediated tear deficiency, topical cyclosporine remains the cornerstone of therapy in dogs and has been shown to improve tear production and ocular surface stability based on established clinical evidence in dogs (28, 29).

Although these therapies have been investigated primarily in dry eye disease, their ability to improve tear film quality, reduce surface inflammation, and support epithelial integrity is mechanistically relevant to corneal ulcer management within this functional domain. Direct evidence in canine corneal ulcer populations remains limited, but ocular surface disease models demonstrate tear film stabilization and epithelial support with these interventions (26, 27).

In addition to pharmacologic stabilization of the tear film, mechanical protection of the corneal surface may further support epithelial recovery. Bandage contact lenses reduce eyelid friction and help maintain a stable tear film environment over the ulcerated cornea, thereby facilitating epithelial migration and epithelialization. In dogs with spontaneous chronic corneal epithelial defects or after epithelial debridement procedures, bandage contact lens placement has been associated with improved healing rates and shorter epithelialization time in several clinical studies (31–33). Potential limitations of bandage contact lens use include microbial contamination, lens displacement, and the need for close clinical monitoring.

Within the present framework, this domain is particularly relevant in non-melting ulcers characterized by delayed epithelial closure, recurrent epithelial defects, or conformational predisposition to ocular surface instability. It may also operate synergistically with regenerative augmentation strategies to improve long-term stability following structural preservation.

By distinguishing ocular surface stability from proteolytic control and regenerative stimulation, the framework emphasizes that durable corneal healing requires arrest of tissue destruction, restoration of epithelial repair, and maintenance of a microenvironment conducive to sustained healing. This domain is considered distinct because epithelial recovery depends on tear film stability, sensory function, and ocular surface homeostasis in addition to cellular repair mechanisms (12–14). Even when tissue destruction is controlled and regenerative pathways are activated, persistent ocular surface dysfunction may compromise long-term healing and contribute to suboptimal functional outcomes (1, 2, 12–14).

### Precision stratification domain (meta-level)

The Precision Stratification domain represents a meta-level component of the proposed framework. Unlike the other domains, it does not constitute a therapeutic intervention. Instead, it encompasses diagnostic and analytical approaches that assist clinicians in prioritizing among domains according to the dominant biological drivers present in an individual case.

Clinical evaluation of corneal ulcers traditionally relies on morphology, depth, and rate of progression. Although these parameters are indispensable for clinical decision-making, they may not fully capture the molecular heterogeneity underlying ulcer progression (1, 2). Advances in tear film analysis and biomarker profiling have introduced the possibility of distinguishing protease-dominant ulcers from those characterized primarily by inflammatory amplification or regenerative insufficiency. Measurement of matrix metalloproteinase activity, assessment of inflammatory mediators, and proteomic analysis of tear composition illustrate potential molecular stratification strategies (1, 2). However, these tools remain largely research-based and are not yet standardized for routine veterinary practice.

At present, most of these approaches remain investigational and are not routinely available in veterinary clinical practice. Accordingly, the Precision Stratification domain should be interpreted primarily as a conceptual and future-oriented component of the framework rather than as an immediately deployable clinical system. In current clinical settings, practical stratification continues to rely primarily on ulcer morphology, stromal depth, rate of progression, cytology, and clinician assessment of ocular surface stability (1, 2).

Parallel developments in computational ophthalmology have expanded the scope of imaging-based phenotyping. Deep learning algorithms applied to *in vivo* confocal microscopy enable differentiation of infectious keratitis subtypes (15). Multi-class slit-lamp image classification systems demonstrate structured corneal phenotyping capabilities (16). Multimodal frameworks integrating imaging features with biomechanical parameters suggest future avenues for objective disease characterization (17). Additionally, AI-assisted analysis of anterior segment optical coherence tomography may enable quantitative monitoring of corneal structural changes (18). Although these technologies were derived primarily from human ophthalmology, they provide a conceptual foundation for species-adapted decision-support systems in veterinary practice.

Within the present framework, precision stratification functions as a decision-support layer that strengthens alignment between pathophysiology and domain prioritization. It does not replace clinical judgment and should not delay immediate structural preservation in time-critical melting ulcers. Instead, it offers a structured means of identifying the predominant biological constraint once acute destabilization has been addressed.

By distinguishing therapeutic action domains from meta-level stratification tools, the framework preserves conceptual clarity while accommodating evolving diagnostic technologies. This distinction also highlights a key research priority: development and validation of canine-specific biomarkers and imaging models capable of translating precision phenotyping into clinically actionable treatment guidance (1, 2, 15–18).

## Discussion

The present framework reorganizes adjunctive management of canine corneal ulcers according to biological priority rather than therapeutic tradition. Instead of centering decision-making on individual interventions, the model emphasizes alignment of treatment emphasis with dominant pathophysiologic drivers. This shift from intervention-centered reasoning to process-centered prioritization is intended to clarify therapeutic sequencing in cases where ulcer progression does not follow a linear or predictable course.

Stromal melting represents the most time-sensitive biological event because structural loss is largely irreversible. Accordingly, the Structural Preservation domain retains primacy when active proteolysis is present (1–3). However, clinical experience indicates that control of matrix degradation does not consistently result in epithelial closure or durable corneal healing (1, 2, 12–14). Delayed re-epithelialization, recurrence, or persistent inflammation may reflect continued activation of upstream inflammatory pathways or inadequately restored surface homeostasis. By situating protease inhibition within a layered biological model, the framework helps explain these discrepancies and discourages reflex escalation of anticollagenase therapy once the dominant constraint has shifted.

Contemporary corneal immunobiology strengthens the rationale for distinguishing inflammatory amplification from matrix degradation. Activation of inflammasome pathways, including NLRP3-mediated maturation of IL-1β, provides mechanistic continuity between pathogen sensing and protease upregulation in microbial keratitis (4–6). NET formation further illustrates how innate defense mechanisms can simultaneously constrain infection and propagate tissue injury (7, 8). These insights support a targeted approach to inflammatory modulation that preserves antimicrobial competence while limiting maladaptive escalation. However, this domain remains supported primarily by translational and experimental data, highlighting the need for controlled canine trials.

The Regenerative Augmentation domain represents a transition from structural rescue toward biologically guided tissue restoration. Platelet-rich plasma offers a clinically accessible method of delivering concentrated growth factors in canine patients (9). At the same time, increasing attention to cell-free regenerative strategies such as secretome and extracellular vesicle-based therapies suggests a transition toward more standardized and scalable biologic products (10, 11). Within the framework, regenerative strategies are not substitutes for structural stabilization but become biologically prioritized once active melting is controlled or when delayed epithelial healing predominates.

The Functional Optimization domain highlights a frequently underemphasized determinant of clinical outcome. Tear film instability, eyelid conformational abnormalities, and neurotrophic compromise may not initiate stromal dissolution, yet they strongly influence whether epithelial repair is sustained (1, 2). Recognition of ocular surface stability as an independent domain clarifies why technically successful structural or regenerative interventions may fail if background instability is not corrected.

A comparative summary of adjunctive therapies, including their framework domain, proposed mechanism, current veterinary evidence base, clinical applicability, and key limitations, is provided in Table 2.

**Table 2:** Classification of adjunctive therapies for canine corneal ulcers according to framework domain, proposed mechanism, evidence base, clinical applicability, and key limitations.

Therapy	Framework domain	Mechanism	Evidence base	Clinical applicability	Key limitations
Autologous serum	Structural Preservation; Regenerative Augmentation	Anti-protease activity, inhibition of collagenolysis, provision of growth factors and epithelial support	[High] Multiple veterinary reports, case series, and long-standing clinical use	Widely available and commonly used in veterinary ophthalmology	Lack of standardization in preparation, storage, and concentration; variable biological composition
EDTA	Structural Preservation	Matrix metalloproteinase inhibition and suppression of stromal collagen degradation	[High] Established veterinary ophthalmic use supported by experimental and clinical studies	Widely available; commonly used for melting corneal ulcers	Limited activity beyond protease inhibition; frequent administration may be required
N-acetylcysteine (NAC)	Structural Preservation	Anti-collagenase and anti-protease activity	[High] Established veterinary ophthalmic use and experimental support	Widely available and inexpensive	Frequent administration required; limited regenerative effects
Doxycycline	Inflammatory Modulation; Structural Preservation	Matrix metalloproteinase inhibition, modulation of inflammatory cytokines, and anti-inflammatory activity	[Moderate] Veterinary and comparative evidence supporting anti-inflammatory and antiprotease effects	Clinically available for systemic administration	Limited ocular-specific clinical studies; potential systemic adverse effects
Platelet-rich plasma (PRP)	Regenerative Augmentation	Delivery of growth factors and bioactive proteins that support epithelial and stromal repair	[Moderate] Limited veterinary clinical studies and case series (9)	Available in selected practices with blood-processing capability	Lack of protocol standardization; variability among preparations; limited controlled studies
Mesenchymal stem cells (MSCs)	Regenerative Augmentation; Inflammatory Modulation	Immunomodulation, paracrine signaling, promotion of tissue repair and regeneration	[Low]Early clinical and experimental studies (10, 11)	Specialized facilities and cell-processing infrastructure required	Small sample sizes, protocol heterogeneity, regulatory considerations, and high cost
Exosomes	Regenerative Augmentation; Inflammatory Modulation	Cell-free regenerative signaling, modulation of inflammation, and enhancement of tissue repair	[Low]Predominantly experimental studies with limited veterinary clinical data (10, 11)	Investigational and not routinely available	Lack of standardization, limited clinical validation, and uncertain long-term safety
Bandage contact lens	Functional Optimization	Protection of the corneal surface, reduction of mechanical trauma, and promotion of epithelial healing	[Moderate] Veterinary clinical studies supporting epithelial healing	Widely available in ophthalmology practice	Risk of infection, lens loss, and requirement for monitoring
Hyaluronic acid lubricants	Functional Optimization	Tear film stabilization, lubrication, and support of epithelial healing	[Moderate] Extensive clinical use; limited ulcer-specific comparative evidence	Widely available and easy to administer	Primarily supportive rather than disease-modifying therapy
Temporary tarsorrhaphy	Functional Optimization	Reduction of corneal exposure and improvement of the ocular surface environment	[Moderate] Established clinical use and long-standing surgical experience	Widely available surgical adjunct	Temporary visual obstruction and requirement for anesthesia or sedation
Third eyelid flap	Functional Optimization	Mechanical protection of the corneal surface and maintenance of a protected healing environment	[Moderate] Long-standing clinical use; evidence largely experience-based	Widely available surgical adjunct	Limited visualization of the cornea during treatment and monitoring

Adjunctive therapies discussed in this review are organized according to the proposed mechanism-based framework comprising Structural Preservation, Inflammatory Modulation, Regenerative Augmentation, and Functional Optimization. The table summarizes the principal biological targets, current veterinary evidence base, clinical applicability, and key limitations of each intervention. This classification is intended to illustrate how established and emerging therapies may be positioned within a biologically ordered framework for canine corneal ulcer management and should not be interpreted as implying equivalent levels of evidence among interventions. Evidence assessments represent qualitative summaries of the literature discussed in this review and are not intended as a formal evidence-grading system.

The Precision Stratification meta-domain extends the framework beyond static categorization. Molecular biomarkers, tear proteomics, and quantitative imaging tools remain largely investigational in veterinary medicine (1, 2). Nevertheless, advances in imaging-based phenotyping and multimodal computational analysis demonstrate how structured categorization of corneal disease is becoming feasible (15–18). Although these technologies were derived primarily from human datasets, they provide a conceptual trajectory for canine-specific validation. The framework does not assume immediate clinical implementation but anticipates future integration of objective phenotyping into domain prioritization.

More fundamentally, the proposed framework provides a conceptual structure for aligning therapeutic priorities with the biological processes most responsible for ongoing tissue loss at a given stage of disease. Rather than focusing primarily on individual therapies or morphological ulcer classifications, the framework encourages clinicians to identify the predominant pathological process, whether collagen degradation, inflammatory amplification, impaired tissue repair, or ocular surface dysfunction, and align therapeutic priorities accordingly. In this regard, the framework functions not as a prescriptive treatment algorithm but as a conceptual framework for biological prioritization.

This prioritization was not based on the historical adoption or popularity of individual therapies. Instead, the domains were ordered according to the biological urgency of the processes they target and the potential irreversibility of tissue loss if those processes remain uncontrolled. Structural Preservation occupies the highest priority because progressive keratomalacia can rapidly result in irreversible stromal loss and globe compromise. Inflammatory Modulation, Regenerative Augmentation, and Functional Optimization address additional biological processes that influence tissue repair, restoration of ocular surface integrity, and long-term maintenance of corneal health. Importantly, the hierarchy is intended to represent relative biological priorities rather than a fixed therapeutic sequence, and multiple domains may require simultaneous attention in individual patients.

The boundaries between the proposed domains should not be interpreted as biologically absolute. Corneal healing is governed by highly interconnected pathways in which inflammatory regulation, extracellular matrix preservation, tissue repair, and ocular surface homeostasis continuously interact. For example, regenerative therapies may exert secondary immunomodulatory effects through cytokine-mediated signaling and macrophage reprogramming, whereas anti-inflammatory interventions may indirectly facilitate epithelial repair by reducing biological constraints on tissue regeneration (19, 20, 25).

Within this framework, prioritization of Structural Preservation reflects not only its role in limiting stromal degradation but also the irreversible consequences of uncontrolled stromal loss. Because active keratomalacia is characterized by ongoing, potentially irreversible tissue loss, suppression of excessive proteolytic activity represents the most time-critical therapeutic objective (1, 2). However, successful structural stabilization should not be equated with the resolution of the entire disease process. By defining Structural Preservation as a distinct biological domain, the framework highlights the limitations of anticollagenase therapy and encourages reassessment of whether the predominant pathological driver has shifted toward inflammatory amplification, impaired tissue repair, or ocular surface dysfunction. In this manner, the framework seeks to reduce reflexive escalation of anticollagenase interventions when healing fails to progress as expected.

The practical utility of the proposed framework remains to be evaluated in representative clinical scenarios, including melting ulcers, indolent ulcers, stromal ulcers, and spontaneous chronic corneal epithelial defects. Given the biological heterogeneity of canine corneal ulcers and the limited availability of controlled canine-specific evidence, prospective assessment of whether domain prioritization improves clinical decision-making, treatment selection, or healing outcomes would help determine the framework’s translational value and guide future refinement (1, 2).

Several limitations warrant acknowledgment. The framework has not yet undergone prospective clinical validation. The framework is derived from narrative synthesis rather than systematic meta-analysis, and canine-specific mechanistic evidence remains uneven across domains. Corneal ulcers represent heterogeneous etiologies, and biological processes often overlap temporally. Therefore, domain prioritization should not be interpreted as rigid sequencing but as a dynamic assessment responsive to clinical evolution. Clinical judgment remains central to this process.

Despite these limitations, the framework provides a coherent structure that integrates proteolysis, inflammatory signaling, regenerative biology, and ocular surface physiology into a unified conceptual model. As a conceptual analysis, its principal contribution is not to provide a comprehensive review of available therapies but to clarify how established and emerging strategies relate within a biologically ordered hierarchy. This structure may assist clinicians in reassessing dominant biological drivers when healing plateaus and may help identify therapeutic priorities that are not readily apparent within conventional severity-based approaches.

At the same time, the degree of clinical validation differs substantially across the therapeutic domains represented in the framework. Structural preservation strategies, including serum, EDTA, and N-acetylcysteine, are supported by long-standing clinical use in veterinary ophthalmology, whereas regenerative approaches such as platelet-rich plasma, mesenchymal stem cells, and exosome-based therapies remain at earlier stages of clinical translation. Consequently, the framework should not be interpreted as implying equivalent levels of evidence among all interventions. Rather, it is intended to provide a biologically organized structure within which therapies can be positioned according to both mechanistic rationale and the evolving strength of supporting evidence.

Advances in precision ophthalmology may facilitate further refinement of this framework and biologically informed patient stratification. Emerging technologies, including tear film proteomics, inflammatory biomarker profiling, immunophenotyping, and artificial intelligence-assisted image analysis, may improve the identification of the principal biological processes driving disease progression in individual patients. Future development of point-of-care biomarkers targeting tear protease activity, inflammatory mediators, or regenerative signaling pathways may enable objective assessment of the biological processes driving disease progression. Together, these approaches could help distinguish ulcers primarily driven by proteolysis, persistent inflammation, ocular surface dysfunction, or impaired regeneration, thereby enabling more targeted therapeutic selection and individualized treatment strategies. Recent advances in AI-assisted analysis of ocular imaging data have further demonstrated the potential of computational tools to support biologically informed clinical assessment (15–18). In parallel, advances in regenerative medicine, including cell-free biologics, extracellular vesicles, and next-generation tissue-engineering approaches, may further expand the range of adjunctive interventions available for canine corneal ulcer management (10, 11). Collectively, these developments may facilitate a transition from morphology-based treatment paradigms toward mechanism-based precision ophthalmology.

## Conclusion

This conceptual analysis presents a mechanism-based hierarchical framework for the adjunctive management of canine corneal ulcers. Interventions are organized into biologically defined domains: Structural Preservation, Inflammatory Modulation, Regenerative Augmentation, Functional Optimization, and Precision Stratification. The model emphasizes temporal urgency and pathophysiologic prioritization rather than therapeutic convention.

Protease inhibition remains the central and time-critical intervention in cases of active stromal melting. However, ulcer progression reflects multiple interacting biological processes involving inflammatory amplification, impaired regeneration, and ocular surface destabilization. Accordingly, adjunctive therapy may be more appropriately guided by the predominant pathophysiologic driver at a given stage of disease rather than by escalation of anticollagenase therapy alone.

The framework is intended to provide a structured conceptual approach that integrates established therapies with emerging insights from corneal immunobiology, regenerative therapeutics, and precision-oriented diagnostics. Evidence strength varies across domains, and canine-specific mechanistic data remain limited. Future studies should define clinically meaningful outcome measures, validate domain-specific therapeutic prioritization, and determine how mechanism-aligned interventions influence corneal integrity, epithelial healing, and long-term ocular surface stability in dogs with corneal ulceration.
